# Supplementary figures and images for: Energy landscapes of peptide-MHC binding
Source: PLoS Comput Biol. 2024 Sep 3;20(9):e1012380. doi: 10.1371/journal.pcbi.1012380 (PMC11398667; doi:10.1371/journal.pcbi.1012380)

**A**

HLA-A\*11:01

Linear model

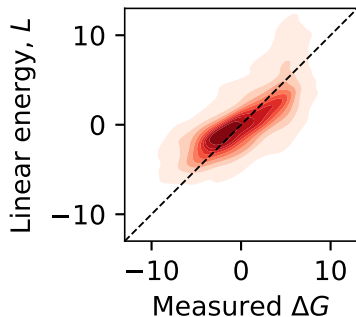**B**

HLA-B\*07:02

Linear model

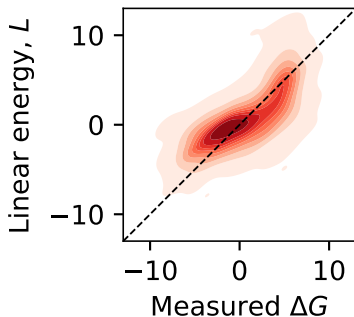

Epistatic model

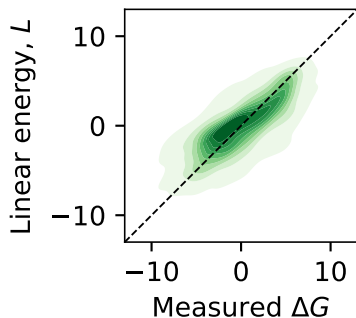

Epistatic model

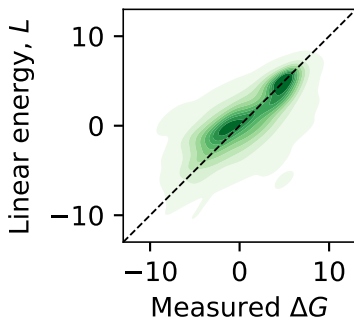

Supplement: S1 Fig — Error plot compares ΔG from validation data and predictions of the linear model (in red), L (dashed line, left). On the right (in green), validation data for ΔG are compared to the global epistasis model (for both alleles, λ = −0.04). The error is computed across 10 cross-validation test sets (contours give densities above 0.01). A: HLA-A*11:01 (Linear model MSE = 5.0, Global model MSE = 4.4) B: HLA-B*07:02 (Linear model MSE = 6.3, Global model MSE = 5.6). (PDF) [file pcbi.1012380.s001.pdf]

**A**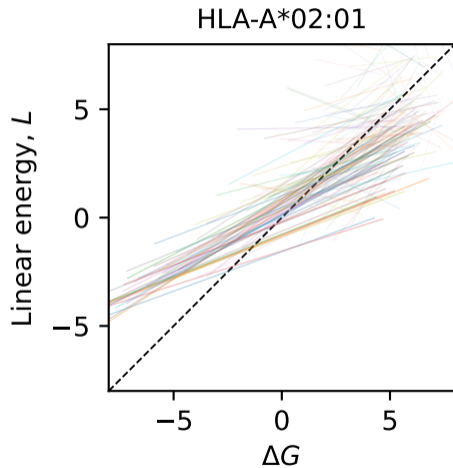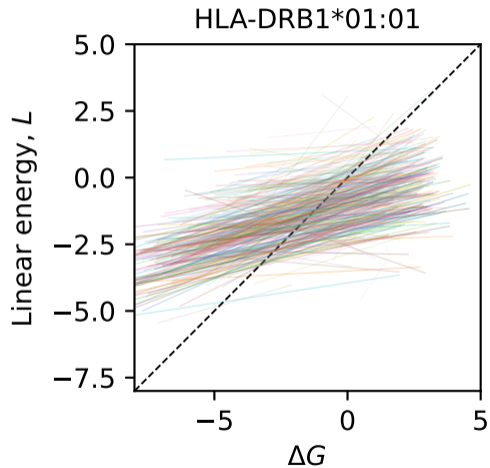

Supplement: S2 Fig — Linear regressions summarising errors of L with respect to ΔG, for each subsample available for HLA-A*02:01 (194 linear fits, on the left) and HLA-DRB1*01:01 (351 linear fits, on the right). Each line is color-coded depending on the start of the L region spanned by the given subsample. The transparency of each regression line is proportional to the number of data points in the given subsample. (PDF) [file pcbi.1012380.s002.pdf]

**A**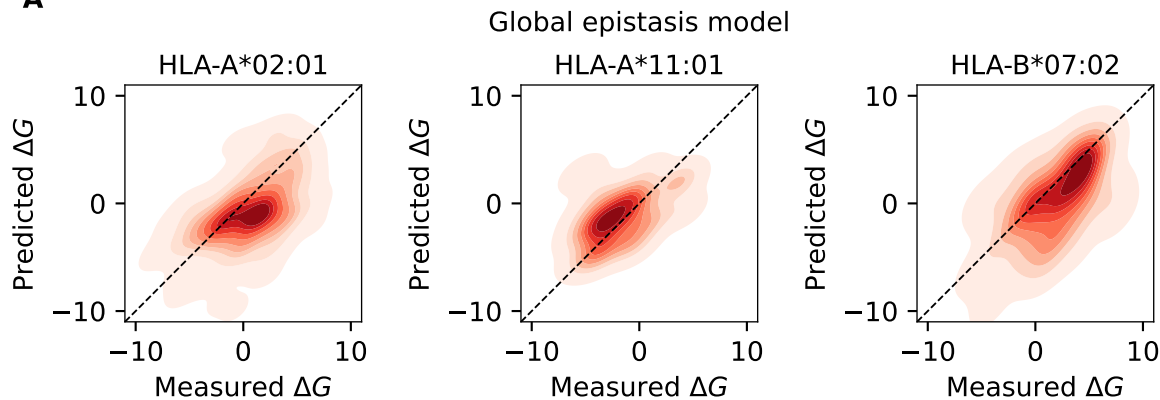**B**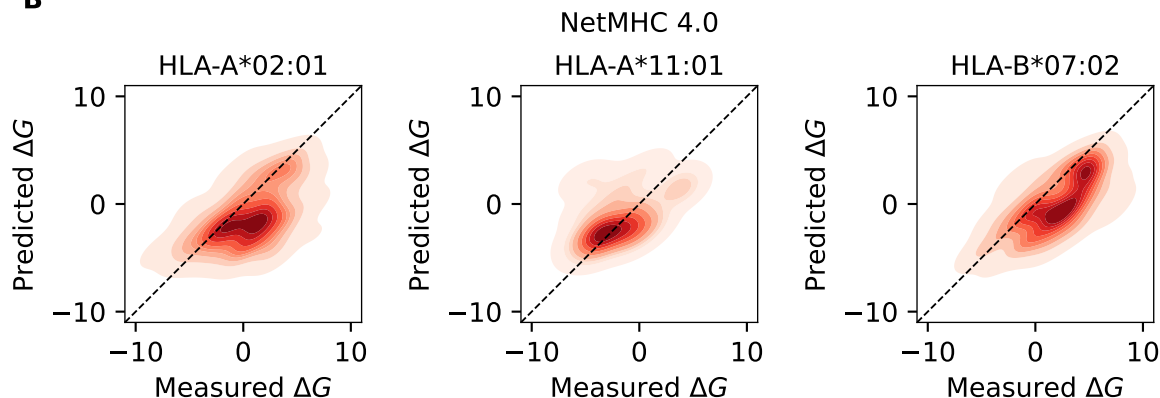

Supplement: S3 Fig — Error plot compares observed energies contained in the blind set, ΔG, and predictions of the global epistasis model with λ = −0.04 for all the tested alleles (A, on top) and NetMHC 4.0 (B, bottom). Contours give densities above 0.01. The blind set contains 465 sequences for HLA-A*02:01, 69 for HLA-A*11:01 and 183 for HLA-B*07:02. (PDF) [file pcbi.1012380.s003.pdf]
