# Supplementary material for: Energy landscapes of peptide-MHC binding
Source: PLoS Comput Biol. 2024 Sep 3;20(9):e1012380. doi: 10.1371/journal.pcbi.1012380 (PMC11398667; doi:10.1371/journal.pcbi.1012380)
Supplement: S4 Fig — Background mutation spectra, shifted to mean 0, for samples of mutations in allele HLA-DRB1*01:01 for strong binders (ΔG ≈ −5, top) and typical binders (ΔG ≈ −1, bottom). A: Spectra computed from the random idiosyncratic model with λ˜=2; B: spectra computed with NetMHCII-2.3. (PDF) [file pcbi.1012380.s004.pdf]

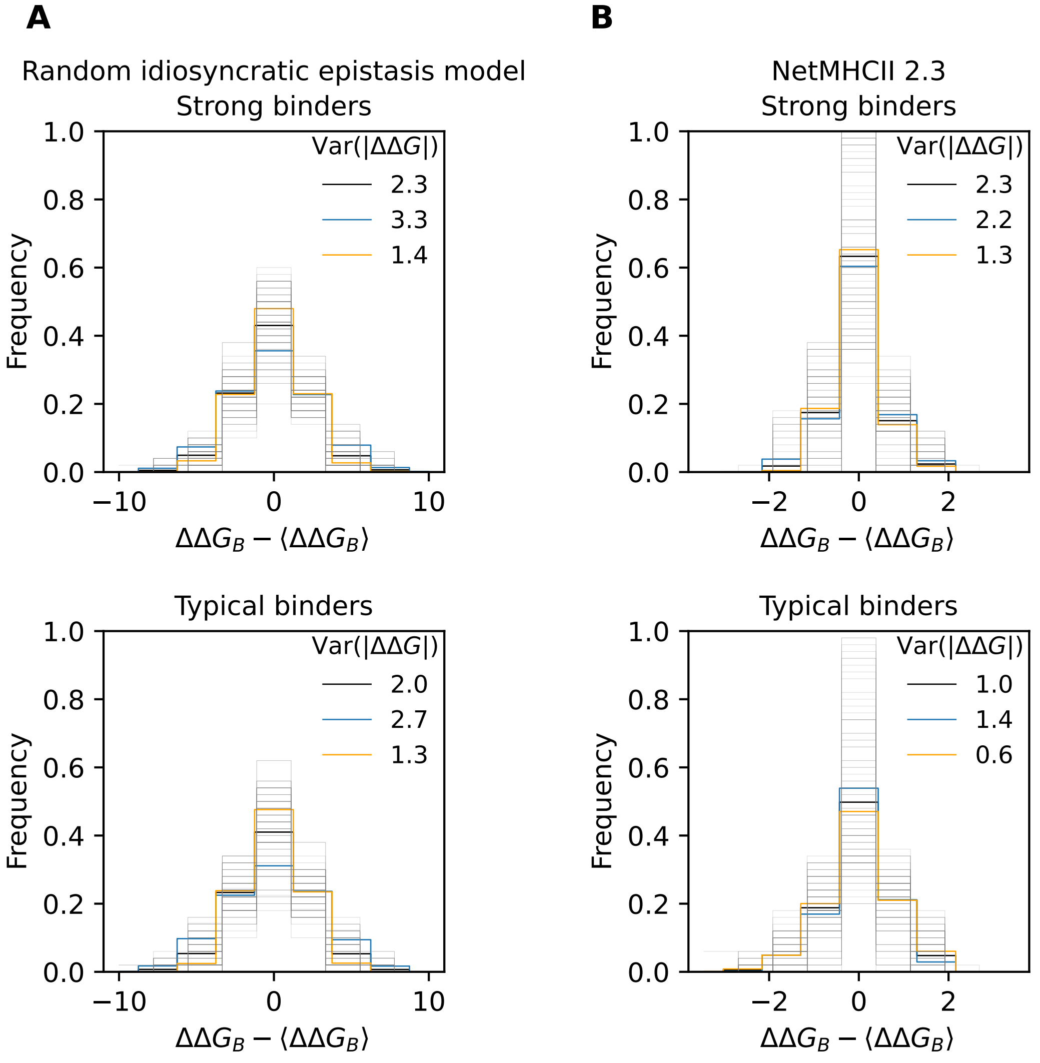
A
B
Random idiosyncratic epistasis model
Strong binders
Typical binders
NetMHCII 2.3
Strong binders
Typical binders
Frequency
Var(|ΔΔG|)
2.3
3.3
1.4
2.0
2.7
1.3
2.3
2.2
1.3
1.0
1.4
0.6
$\Delta\Delta G_B - \langle \Delta\Delta G_B \rangle$
