# Supplementary material for: Energy landscapes of peptide-MHC binding
Source: PLoS Comput Biol. 2024 Sep 3;20(9):e1012380. doi: 10.1371/journal.pcbi.1012380 (PMC11398667; doi:10.1371/journal.pcbi.1012380)
Supplement: Attachment — Submitted filename: Rebuttal_letter.pdf [file pcbi.1012380.s014.pdf]

Dear Dr. Piero Fariselli, Dear Dr. Nir Ben-Tal,

Thank you and the reviewers for considering our manuscript. We appreciate the constructive comments and the overall positive feedback. In this revised version, we address all the comments comprehensively. The major changes are listed below:

1. In response to the question of reviewer 2, we have introduced the analysis of HIV-derived MHC ligands, extending the section 'Immune escape evolution' and showing the results in Figure 5C. Here, we show how in MHC-I single mutations derived from HIV peptides tend to preserve the binding quality when occurring in non-anchor positions, while escape mutations are more frequent in anchor positions. Conversely, escape mutations in MHC-II binding cores are distributed broadly throughout anchor and non-anchor positions.

2. In response to the suggestion of reviewer 2, we have added Figure S3 to the main text, as Figure 3. Here, we show the effect of global and idiosyncratic epistasis in simulated landscapes, reproducing the pattern observed in the data (Figure 2 and Figure S2).

3. In response to the suggestion of reviewer 1, we have extended the abstract, the author summary, and the discussion section, adding future research directions and more context to our findings in the field of Immunology.

Please find below a detailed response to all reviewer's comments.

Best regards,

Laura Collesano, Marta Łuksza and Michael Lässig

Reviewer 1

The authors have attempted to design computationally sequence-dependent energy landscapes of MHC-peptide binding encode class-specific nonlinearities (epistasis). MHC-I has a smooth landscape with global epistasis; the binding energy is a simple deformation of an underlying linear trait. This form of epistasis enhances the discrimination between strong-binding peptides. This study offers a fascinating insight into the intricate world of peptide-MHC binding and its role in immune recognition. The distinction between MHC-I and MHC-II energy landscapes sheds light on their respective complexities. The research underscores the significance of sequence-dependent energy landscapes in peptide-MHC binding and their implications for T cell immunity. It's intriguing to see how MHC-I exhibits a smoother energy landscape with global epistasis, while MHC-II displays a more rugged landscape with idiosyncratic epistasis. The utilization of datasets from the Immune Epitope Database adds credibility to the findings, ensuring a robust analysis of peptide-MHC interactions. The study's approach in inferring linear energy models and exploring epistatic landscapes provides valuable insights into the mechanics of immune recognition. The comparison between linear and global-epistasis models elucidates the suitability of different modeling approaches for MHC-I alleles. The performance evaluation against NetMHC 4.0 highlights the efficacy of the proposed global-epistasis model, particularly for MHC-I alleles. Having said that, the paper could still be a good reference for similar studies, and thus, I recommend its publication after the author addresses all the major and minor concerns satisfactorily.

Thank you for the detailed review of our manuscript and the positive and encouraging feedback. We have addressed all the major and minor concerns. The detailed answers can be found below.

- Abstract: The abstract provides a clear overview of the study; however, it would benefit from a brief mention of the significance of the findings and their potential implications in the field of immunology.

  We have enriched the abstract with additional mentions of the significance of the findings and

potential implications for the field of immunology.

- Introduction: While the introduction sets the stage for the study, it lacks a clear statement of the research gap or question addressed by the study. Providing a more explicit statement of the problem would enhance the introduction.

  A clearer statement on the research gap has been added in lines 44-48.

- Methods: The study's methodology for dataset curation ensures the reliability of the findings by incorporating quality control measures. It's impressive to see how the research integrates experimental data with computational modeling to unravel the complexities of peptide-MHC interactions. The comparison between linear and epistatic models highlights the importance of considering higher-order interactions in accurately modeling peptide-MHC binding. Overall, this study significantly contributes to our understanding of immune recognition mechanisms and has implications for the development of novel therapeutic interventions. The study's comprehensive approach, encompassing experimental data analysis and computational modeling, ensures a thorough exploration of peptide-MHC binding dynamics.
  The methods section is detailed and well-structured, providing clarity on data collection, analysis, and model optimization. However, including information on potential limitations of the methods or assumptions made during the analysis would improve the section's comprehensiveness.

  We have added additional information on the model assumptions and limitations in the Method sections. These include the extraction of the MHC-II cores, the model optimization, and the data and code availability.

- Results: The results section presents key findings regarding the energy landscapes of MHC-peptide binding. Adding more contextualization of the results within the broader research landscape would enhance the section's readability.

- Figures and Tables: The figures and tables are informative and well-designed. However, providing more detailed figure legends and ensuring consistency in labeling throughout the manuscript would improve clarity.

  The figure labels and legends have been adjusted to ensure consistency, especially between the main text and supplementary information. We have modified the table caption to provide a more detailed explanation of the content.

- Discussion: The discussion should not only summarize the findings but also provide insights into their significance and potential impact on the field. Additionally, discussing any unexpected results or limitations of the study would add depth to the discussion.

  Thank you for your comment. We added a statement about the limitation of our model in lines 445-447. In the following lines, we broadened the discussion with further insights on the potential impact of our work on further research on Biological Macromolecules and Immunology.

- The analysis of mutational effect spectra offers a deeper understanding of the impact of mutations on peptide-MHC binding, especially in the context of escape evolution. The observation of modularity in presentation and T cell recognition underscores the complexity of immune evasion strategies employed by pathogens. The study's methodology, including dataset curation and model optimization, ensures rigor in the analysis of peptide-MHC interactions. It's noteworthy how the study addresses the challenge of complexity in modeling peptide-MHC interactions, particularly in the case of MHC-II. The simulations of random energy models provide a useful tool for understanding the underlying mechanisms driving peptide-MHC binding.

  The research contributes to our understanding of immune escape evolution by highlighting the distinct mechanisms employed by MHC-I and MHC-II. The findings regarding the distribution of large-effect mutations offer valuable insights into the vulnerabilities of antigenic epitopes to

escape mutations. The study's focus on the interplay between sequence-dependent energy landscapes and immune recognition provides a holistic view of the immune system's functioning. The exploration of different regimes of epistasis enhances our understanding of the adaptive strategies employed by pathogens to evade immune detection. The analysis of energy statistics underscores the importance of considering both strong-binding antigenic epitopes and typical binders in studying immune escape evolution.

We thank you for the detailed summary of our analysis, as well as for your positive feedback.

- Language: The manuscript generally adheres to academic writing standards. However, some sentences are overly complex and could be simplified for better readability. Additionally, ensure consistency in terminology and avoid jargon when possible.

- Grammatical errors: The manuscript contains several grammatical errors and awkward sentence constructions. A thorough proofreading and editing process is necessary to correct these issues and enhance the clarity of the text.

  As pointed out, several grammatical errors were found. We have edited and proofread the manuscript, increasing its readability. Additionally, we have simplified some overly complex sentences.

- Clarity of Presentation: The manuscript would benefit from clearer transitions between sections to guide the reader through the logical flow of the study. Additionally, ensuring that each paragraph focuses on a single idea would improve readability.

- Citation: Ensure that all references are cited correctly and consistently throughout the manuscript, following the appropriate citation style guidelines.

  Thank you for your suggestion. We revised the citations and new references were added, in particular: ref. [27] in line 38, ref. [28] and ref. [30] in line 41, ref. [51] in line 148.

- Statistical Analysis: Provide more detailed information on the statistical methods used, including assumptions made and measures of uncertainty or variability.

- Future Directions: Conclude the manuscript with a brief discussion of potential future research directions or avenues for further exploration in this area. The conclusion should reiterate the main findings of the study and emphasize their importance in advancing knowledge in the field of immunology.

  We have added a short statement on the future directions of our work in lines 453-456.

- Graphical Abstract: Consider creating a graphical abstract to visually summarize the key findings of the study and attract readers' attention.

- Impact Statement: Include a brief impact statement highlighting the significance of the study findings and their potential implications for research, clinical practice, or policy development.

  We have improved the Author Summary by adding more context, including a sentence on the significance and implications of our work.

- Missing references: https://doi.org/10.1038/s42256-022-00459-7;

  This reference is now in line 38.

- Final Remarks: Final remarks summarizing the key points addressed in the review and expressing confidence in the manuscript's potential for publication with revisions. I would like to see the revised version of this manuscript.

Reviewer 2

In this paper, the authors fit models of varying simplicity to recapitulate data on peptide binding to human HLA molecules. This task is currently performed by NETMHCpan which uses a neural network trained on the same data as the authors use in this paper. However, this paper sheds new light on peptide binding to MHC. The main result is that a simple multiplicative quadratic model (global epistasis) can fit the data for peptide binding to MHC I molecules as well as NETMHCpan with far fewer parameters; i.e., for MHC I neural nets are an overkill. The authors also show that for MHC II molecules more complex models are required and the use of neural nets for obtaining accurate models is justified. These results make intuitive sense as the MHC I groove is closed at the ends with strong dependence of peptide binding on the two key anchor positions (2 and 9). On the other hand, the MHC II groove is open at the ends and the binding register can shift for different peptides binding to the same class II allele. But, to my knowledge, the authors are the first to demonstrate this quantitatively. Therefore, I recommend publication of the paper in PLOSCB after the reviewers have a chance to revise the manuscript to address the points noted below.

We thank the reviewer for the encouraging comments and the positive assessment. The points raised helped clarify the presentation of key concepts of our work and enriched the results section, as well as the discussion, adding more depth to our findings.

Specific points:

- Why is ΔS a measure for how good a linear model would be? For example, ΔS could be large for class I molecules because the value at the 2 and 9 positions are very high. I could not understand why this pertains to how good a linear model will be.

  The construction of ΔS in line 110, assumes the independence of each column's contribution to the total energy. Moreover, it estimates the binding motif learnability from the amino acid sequence. From the earlier assumption, the total ΔS estimates how much information about binding energy/motif can be contained in an *additive* energy matrix, inferred from the data. For MHC-I, due to the higher information gained from the motif, we conclude a linear model has the power to explain the data. Vice versa, the reduced information content displayed by MHC-II shows that the information about presentation cannot be in the additive sequence motif alone.

- There is a syntax problem on line 451.

  The sentence has been rewritten, now in line 481.

- In lines 139-144 it is first noted that the likely factors for bad agreement for MHC II with the linear model are variation in binding cores and additional interactions from flanking regions. But then it is noted that epistasis is the cause. Some clarity is required here.

  In the context of this work, the term epistasis refers to a general nonlinearity shaping the energy landscape of peptide-MHC interactions. However, it must find an origin in the biological mechanisms underlying the binding event. In these lines, we tried to formulate a hypothesis on the origin of idiosyncratic epistasis in MHC-II, finding good candidates on the sliding mechanism of the peptide in the binding pockets, together with the poorly understood role of the peptide flanking regions. All of these contribute to the nonlinearity observed in the data, referred to as epistasis.
  We clarified this concept in lines 145-150.

- For class I molecules, would the fit be of poorer quality than that obtained using Eq. 3 if the interactions between the amino acids of the peptide and the HLA molecule were taken to be those in the Miyazawa-Jerningan (MJ) matrix that is used to model protein folding?

We thank the reviewer for the interesting question. The use of the Miyazawa-Jernigan (MJ) matrix for tackling this problem can be difficult for the following reasons. Firstly, the binding pocket of HLA-I is a 3D structure. The residues interacting with the peptide have been identified in the work 'The pockets guide to HLA class I molecules', Biochem Soc Trans 2021. However, it is unclear how these residues interact to give an effective binding energy for different alleles. We do not exclude the existence of a detailed contact map, which describes the interaction between one or more HLA residues and the peptide positions.
In first approximation, we assume the prevalence of a one-to-one interaction between HLA and the peptide. The goodness of this assumption could be tested by computing the Kullback-Leibler divergence (ΔS) between the MJ matrix and single columns of our inferred linear energy matrix. The results of this comparison are reported in the Figure below. Here, we show how for less informative positions each MJ matrix column scores similar ΔS values, suggesting the existence of more intricate interactions involving several residues. Despite the decreased variability, also for more informative positions (2 and 9) we cannot identify a single column that minimizes ΔS. This increases the difficulty of the comparison, given the necessity of inferring

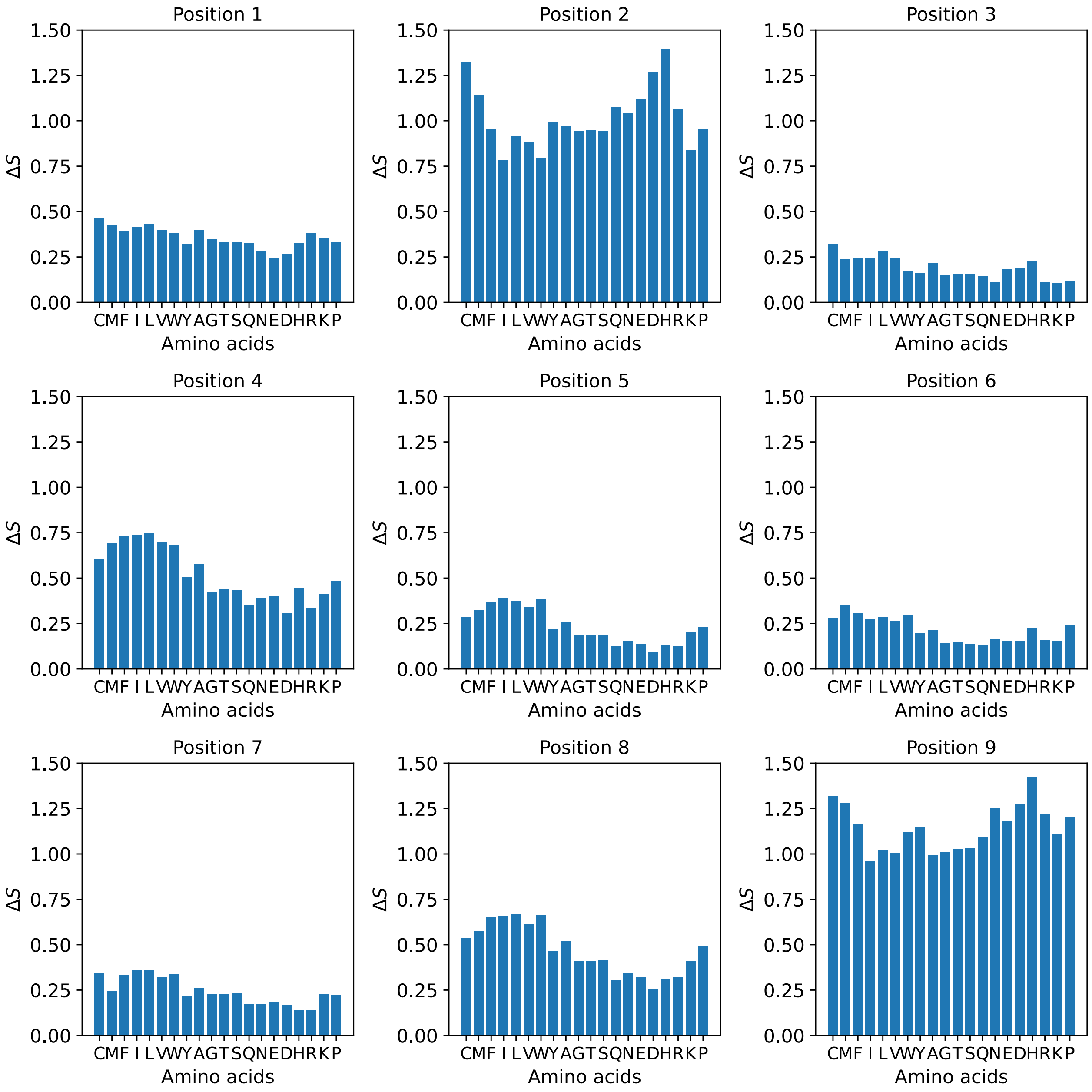

Figure 1. Position-specific Kullback-Leibler distance (ΔS) computed comparing the MJ matrix to single columns of the inferred linear energy matrix.

weights for multiple contact residues. Moreover, the weights inference for the central positions may be less precise and less significant due to their less informative nature.
Previous studies have pursued this analysis using crystal structures of pMHC complexes, as in the work: 'Structure-based prediction of binding peptides to MHC class I molecules: Application to a broad range of MHC alleles', Protein Science 2000. However, an additional difficulty in this approach lies in the existence of population-wide mutations in the HLA genome. These mutations can lead to different residues in the binding pocket and influence the construction of the equivalent matrix.

- What is meant by "original sequence" on line 163?

  Here, we use the term 'original' when referring to the 'wildtype' sequence, in the context of the calculation of the mutational effect $\Delta\Delta G$. However, we recognize that the term may be misleading. Therefore, it has changed to 'wildtype', now in line 169.

- I suggest that Fig. S3 be moved to main text as there is a whole section describing it in the main text and it is informative.

  Figure S3 is now in the main text as Figure 3. Compared to the previous version contained in the supplementary information, we formatted the plot size and labels to match more carefully the main text style.

- The acronym BIC should be defined in Fig. 3 – the acronym is never defined in the main text where it is written as Bayesian information criterion.

  We define now the acronym in the figure caption of the new Figure 4 (old Figure 3).

- Is there sufficient data for escape mutations due to loss of MHC presentation during HIV infection to see if they are largely concentrated at anchor positions for MHC I and distributed all over for MHC II? This would be consistent with the authors' findings, and if so, the paper would be strengthened.

  Thank you for the interesting question. As suggested, we have performed an analysis on HIV-derived MHC ligands. Despite the presence of some HIV sequences in our testing and training data, we agree that further analysis of these epitopes strengthens our findings. Therefore, we have included it in the paragraph: 'Immune escape evolution' and in an extended version of Figure 5.
  In Figure 5C, we show that inferred escape mutations from MHC-I binding are more frequent at anchor positions. For MHC-II, these mutations are more broadly distributed, which is the expected pattern described by idiosyncratic epistasis. More details are described in a dedicated section in Material and Methods, in lines 545-549.

- Define the meaning of physiological binding threshold in line 98 better as it sets the scale for ΔG.

  The physiological binding threshold introduced to set the scale for ΔG, now in line 101, is a conventional value used to discriminate between binders and non-binders in analogous studies, as the one in Ref. [43]. However, in Ref. [42], it is defined as the concentration in nM able to elicit a T cell response.
